# ‘I praie ye send for the courall’: children’s coral as the physical embodiment of parental hopes and fears in early modern England

**DOI:** 10.1080/1081602X.2024.2360935

**Published:** 2024-06-07

**Authors:** Francesca Elizabeth Richards

**Affiliations:** Centre for Medieval and Early Modern Studies, University of Kent, Canterbury, UK

**Keywords:** Children, coral, emotions, materiality, protection

## Abstract

Mediterranean red coral has long been believed to be imbued with sacred, spiritual and healing power and was given to children across Europe in the form of an amulet, teether or medicine. In early modern England, portraits of children from affluent families and medical receipts provide snapshots of coral usage, a moment in time, which do not adequately reveal the spiritual, therapeutic and affective significance of this material within the context of unique family circumstances and a multitude of perceived threats to children’s health. Focusing on five case studies, this article delves into the lived experience of families who deemed coral objects an essential childhood accessory in the period from 1590 to 1775. Julian Herrick, a goldsmith’s widow, Sir John Oglander, a knight caught up in the civil war, Dr Garencières, a doctor confronting dangerous infection, Lady Blackett, a devoted grandmama, and Ann Lord, a woman facing destitution, all placed value on coral for their young children. This study draws on a diverse range of material and documentary evidence from the seventeenth and eighteenth century to unpack the significance of red coral for parents within a changing religious, political and medical landscape. Building on scholarship within material culture studies and the history of emotions, this study also situates children’s coral objects within emotional relationships and seeks to uncover the implicit meanings such objects held which could not be written or articulated due to illiteracy, controversy or persecution. By combining a micro-historical approach with a broader thematic analysis, the case studies presented indicate that coral occupies an important place in the history of the early modern family as the embodiment of the anxieties of parents and grandparents, a means to soothe and protect young children and a material expression of hope, love and faith, particularly during periods of crisis and separation.

## 1 Introduction

For parents of young children in 21st century Britain, common worries are teething pains, fevers, incessant crying and the risk of sudden infant death syndrome. Amongst our domestic armoury, you might find a brown glass bottle of Calpol (a popular brand of infant fever suspension), a plastic teething toy and an electronic baby monitor. This combination of tools to relieve symptoms and safeguard infant health is a familiar and socially acceptable way to care for children at home, with recourse to an Accident and Emergency Department a reassuring option in times of crisis. In early modern England, parents shared similar anxieties about infant teething, fevers, or death by being ‘overlaid’ by a drowsy caregiver. The inauspicious position of the planets at birth or the suspected Evil Eye of a witch might also present causes for concern. With such an array of potential threats to child health and an infant mortality rate considerably higher than it is today, infancy could be an extremely anxious time for parents.

This article explores how and why red coral, an imported natural material, held an important place in the domestic armoury of English families in the early modern period. Since Antiquity, red coral was harvested from the Mediterranean sea and perceived to have special properties particularly beneficial to children. According to Greek myth, red coral was created from the blood of Medusa’s severed head and imbued with apotropaic power to deflect the Evil eye (Ovid, 1985, p. 161, l.740–52). Pliny the Elder, in his encyclopaedic work *Natural History* (c.77 A.D.), wrote ‘branches of coral, worn as an amulet by babies, are believed to be protective’ and coral powder could be taken with water to treat stomach spasms and fevers or applied externally in a salve (2004, p. 281). Here Pliny the Elder presented coral as a treatment for both supernatural and natural ailments.

This article examines how red coral offered a means for parents across the social hierarchy and confessional identities to negotiate both enduring and new concerns about the wellbeing of their offspring. I argue that red coral given to children by anxious parents acted as the material expression of a desire to protect the child and hopes and fears regarding their child’s future. A material focus on red coral builds upon the documented anxieties of parents recorded in diaries and correspondence and considers what could not be written or was difficult to verbally articulate (Styles, 2010, p. 70).

Coral could materially embody the concerns of parents who were living in challenging circumstances. The case studies analysed here include two landed gentry families from the North and South of England, a family of upwardly mobile, affluent goldsmiths from Leicester, a physician working in York and London and a woman from the Spitalfields weaving community. Whilst these examples demonstrate variety in class, trade, education and location, they represent the diverse landscape of early modern England in more interesting ways. As Royalists, Catholics, proponents of lapidary medicine and astrology and individuals affected by suicide and desertion, these parents were living in contravention of dominant ideologies during periods of religious strife, political conflict and intellectual disputes about medicine, a healthy body and soul and the ideal family. Rather than encountering security and stability, these cases illuminate coral’s place within tension, fear and tragedy.

The term ‘parents’ is broadly conceived here, recognising the concerns felt by grand-parents and godparents within extensive kinship networks and, by contrast, the experiences of lone and deserted parents. Within these case studies there is a recurring theme of distance and separation, as parents and grandparents were separated from young children due to wet nursing practices, separate family residences, the demands of war, premature death, or, as in the last example, fostering by an institution. Intended to be constantly worn by children, coral objects may be understood as safeguarding the child *in loco parentis*. Red coral thus became a material imbued not only with meaning but also emotion, an animated object intended to convey love, preserve health and lend protection while serving as a reminder of the multiple natural and supernatural threats to the innocent recipient.

## 2 Coral and children in Europe

Coral has been identified as an important component of jewellery, medicines, amulets, and teethers and an indicator of status amongst privileged families in Europe. Previous research on the perceived benefits of coral has been conducted within the fields of art history and the history of medicine. Ronald Lightbown (1992, pp. 90–92, 207, 236), Marion Endt-Jones (2013) and Marcia Pointon (2010, pp. 127–144) have considered coral’s rich symbolism within European culture as a material imbued with mythical, religious and healing power. Scholars including Maria Do Sameiro Barroso (2017), Christopher Duffin (2013, 2022) and Nichola Harris (2009, 2022) have analysed coral’s medicinal uses in the medieval and early modern world, tracing coral in medieval lapidaries, mendicant texts and physicians’ treatises attributed to Arabic, African and European authors such as Avicenna, Constantinus Africanus, Albertus Magnus and Petrus Hispanus through to the works of early modern medical writers including Thomas Phaer, Thomas Willis, and John Pechey. Coral was believed to be particularly useful in treating children suffering from convulsions, epilepsy, teething and anaemia and was widely available in apothecary shops in the seventeenth century in simple and compound medicines (Duffin, 2013, 2022; Endt-Jones, 2013; Harris, 2009, 2022).

The classical belief in the apotropaic properties of red coral survived in Renaissance Tuscany. Jacqueline Musacchio describes affluent parents adorning their infants with red coral amulets to protect against the dangers of illness, suffocation, accident or evil spirits. This was particularly important for those sent out of the family home to wet nurses (2005, p. 151). When small branches of red coral were harvested in the Mediterranean Sea, these were deemed particularly suitable for children and were carefully accounted for by elite families as an essential childhood accessory. Even the Christ Child was adorned with coral beads in Renaissance portraiture, associating coral with childhood in Catholic iconography. Coral beads or branches might also be strung alongside animal teeth or with sacred devotional items such as *Agnus Dei* pendants, small round tablets stamped with the image of the lamb of God (Musacchio, 2005, p. 152).

Red coral was employed as both a lapidary medicine and a symbol of love and family prestige for elite children in the Netherlands during the Dutch Golden Age. Portraits commonly depicted children either wearing coral necklaces and bracelets or holding coral teethers (Kuus, 2000a, p. 79). Saskia Kuus suggests parents adorned their young children with coral beads to prevent convulsions and sooth nerves, as recommended in the Dutch herbal, Dondanaeus’ *Cruydt-Boeck* of 1554 (2000b, p. 102). Coral was also used in children’s *Rinkelbels*; silver or gold rattles with whistles, bells and a teether. Annemarieke Willemsen notes that expensive and ornate *rinkelbels* gifted by a significant relative or godparents served as an indicator of the family’s status and were handed down as a family heirloom (2000, p. 65). While *rinkelbels* could incorporate teethers made of rock crystal or a wolf’s tooth, Wozny argues that the choice of coral branches symbolised the devotion of parents and children, drawing on Old Testament references to parents as branches or vines and children as beloved fruit (2015, p. 101).

**Figure 1:**
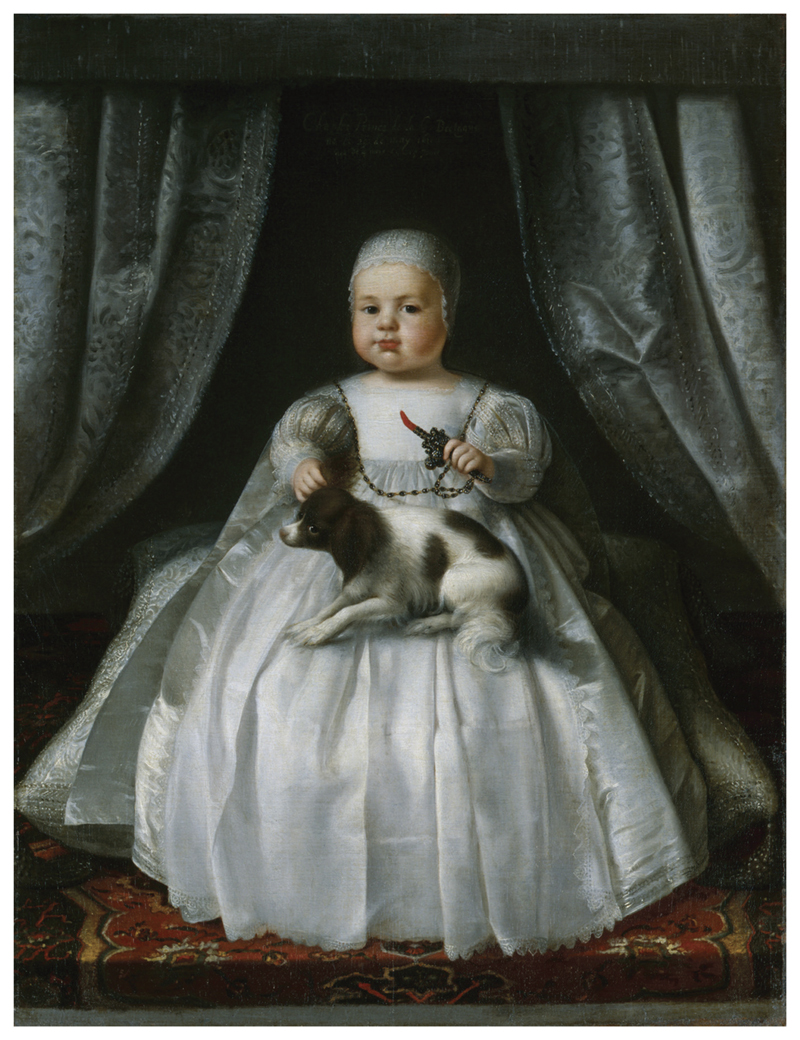
Unknown artist, (1630). King Charles II, Oil on canvas [NPG 6403]. National Portrait Gallery, London, United Kingdom.

The popularity of red coral in Post-Reformation England had parallels with the Dutch usage as children’s beads, teethers and medicine. People travelled between London and Amsterdam with ease, as the Netherlands and England, both Protestant countries, enjoyed rich intellectual and economic exchange. Affluent English children, like Dutch children, were depicted in portraits wearing coral beads and teethers throughout the early modern period. Painted in 1630, a chubby and robust Charles II, aged 4 months old, held a beautiful example of a coral teether set in silver (see Figure 1). Marcia Pointon has described coral jewellery and teethers as ‘luxury products’ which English aristocratic parents gave to their children as a form of protection and symbol of status (2010, p. 130). However, while the ownership of coral by elite children is certainly more visible in portraiture and extant coral teethers set in precious metals seem to support this view, there is evidence that coral was used more widely and could be considered an ‘everyday’ material within the English home.

## 3 An everyday material

Coral was a familiar material within early modern England, demonstrated by its frequent appearance in a range of sources including medical manuals, portraits, domestic receipt books and family correspondence as well references to it being popular or highly regarded in texts. Coral was well-established within English medical receipts for children. Thomas Phaer, whose short remedy book *The Boke of Chyldren* (1544) was intended for a broad audience, regarded coral’s benefits as uncontroversial. He remarked,

I am content to declare thys of corall: that by consent of all authors it resisteth the force of lightenynge, helpeth the children of the fallynge evyll, and is very good to be made into pouder and drunken against all manner of bleeding of the nose or fundament. (1999, p. 52)

A century later Nicholas Culpeper, apothecary and herbalist, deemed coral to be so important for newborns as a prophylactic it must be given at birth. He advised parents in *A physical directory* (1651):

If ten grains of red Coral be given to a child in a little breast-milk so soon as it is born, before it take any other food, it will never have the falling sickness, nor convulsions. The common dose is from ten grains to thirty … red Coral preserve the body in health, and resist fevers. (1651, p. 35)

Here Culpeper described coral as a universal preventative for two of the most worrying conditions for parents regarding infant health; fits and fevers.

The writings of Reginald Scot (1538–1599) and John Aubrey (1626–1697) indicate popular usage of coral as an amulet was very common in England over a long period. Scot, a Kent gentleman and Member of Parliament, wrote in *The Discoverie of Witchcraft* (1588),

The corrall preserveth such that beare it from fascination or bewitching, and in this respect they are hanged about children’s necks. But from whence that superstition is derived … I know not: but I see how readily the people are to give credit thereunto, by the multitude of corrals that waie emploied. (2020, p. 166).

Scot referred to the use of coral as both widespread and a mere ‘superstition.’ A century later John Aubrey, antiquary, natural philosopher and folklorist wrote in his *Miscellanies* (1692) that coral was used against the Evil Eye, ‘Coralls are worne by Children still. The Irish doe use a Wolves fang-tooth set in silver and gold for this purpose; which they hold to be better than Coral’ (1972, p. 233). According to Aubrey, belief in the Evil Eye was very much still prevalent, with coral amulets continuing to play an important apotropaic role in England. He noted the Irish preferred wolves’ teeth; whether this was because animal teeth were cheaper, easier to obtain or believed to be more effective than coral is unclear.

From these examples, rather than simply categorising coral as ‘exotic’ or a ‘luxury’, we can broaden our understanding of coral as a popular material that held complex meanings and transcended social hierarchies. Accessing these meanings poses certain challenges. As Karen Dannehl has stated, the problem with everyday objects is that their meanings are by nature implicit, too obvious to mention (2018, p. 175). It is only when they are deemed deserving of comment that they become important and are lifted out of the mundane. While understanding exactly why coral was considered essential for children is difficult, the fact that a child’s ‘coral’ was judged worthy of mention in family correspondence suggests it held significance beyond the humdrum world of the domestic. However, because coral’s purpose is rarely made explicit amongst a range of possibilities, it requires deeper contextual analysis. Indeed, it seems common to have referred simply to ‘the coral’ which does not detail its form as a teether, jewellery or medicine, or its intended purpose. In the five case studies presented here, only Theophilus Garencières stated the type of coral object, its express purpose and emotional significance. Indeed, coral is at once exotic and domestic, special and everyday, a commodity and a talisman. The gifting of coral to the very young may assert its significance within emotional and spiritual worlds, challenging the narrative of early modern consumption as a process of ‘despiritualisation’ and ‘commodification’ (Pennell, 2018, p. 236).

## 4 Material approach

This article presents an alternative analysis of coral’s importance within the early modern family by moving beyond disciplinary boundaries or teleological ideas of secularisation. Downes, Holloway and Randles have proposed in *Feeling with Things* that we can conceptualise materials or objects in multiple directions, as ‘potent’, ‘binding’ or ‘moving’ (2018, p. 5). We can interpret coral using all three of these categories, as imbued with spiritual and occult power, as significant in affective bonds and gift giving, and travelling in space and time as a foreign import and accessory travelling with and between children and across generations.^1^ A material focus on coral and its place in early modern family life reveals a material at once familiar, domestic and viewed as highly potent within multiple cosmographies. I define cosmography here as the perception of forces active within the universe. During this period, these forces might be understood as the power of the divine as interpreted within Protestant, Catholic or sectarian doctrines, the influence of the movement of the planets and stars or the malign intentions of evil spirits or witches. Rather than the ‘decline of magic’ as put forward by Keith Thomas (1973) I argue that the significance of coral to early modern families suggests people drew on multiple cosmographies which co-existed rather than replaced each other. This builds on work by Ivanic (2021) on evidence that burghers in early modern Prague lived in a rich, spiritual world where devotional items and ‘lived religion’ could not be neatly categorised according to confessional identities.

Historians of emotions offer ways to interrogate coral as a material operating within emotional relationships. Barbara Rosenwein presents the concept of ‘emotional communities’ in her research on the Middle Ages, defining such a community as ‘a group in which people have a common stake, interests, values and goals’ or even a shared ‘discourse’ (2006, pp. 24–5). We might consider the families in the case studies analysed here to constitute an ‘emotional community’ despite including parents and grandparents of both genders, in diverse geographical locations, with different confessional identities and political allegiances and varied levels of social status and financial security. Hannah Newton argues that diaries and letters in early modern England reveal commonly held parental emotions of joy, anxiety, distress and unbearable grief (2012, pp. 121–157). Parents thus constitute an ‘emotional community’ in terms of similar aspirations and concerns for their children. The fears they held might not be articulated, expressed or even consciously acknowledged and drew on diverse understandings of health, religion, status, the natural and the supernatural. Yet fundamentally, during this period of high infant mortality, the primary concern which united these families was the basic desire for their child’s survival.

While Rosenwein focuses on written sources, material culture offers an alternative way to access parental values and emotions. Within each family, coral accompanied the child through infancy as a necklace, bracelet or as a teether worn on a ribbon. It might be handed down between siblings and generations. Sherry Turkle describes ‘evocative objects’ as ‘companions to our emotional lives or as provocations to thought’ (2011, p. 5). The handing down of coral to children within early modern family networks rendered coral a cross-generational ‘companion’, presenting continuity of love and care towards the family offspring, ‘a marker of relationship and emotional connection’ essential during periods of separation. As a ‘provocation to thought’, coral could act as a reminder of the threats to child well-being, a call to protect the child from pain and contagion, perhaps also the unseen and the unsaid, the influence of the moon, the Evil Eye. A coral object might also serve as a memento of the child or their childhood. Possessions have a role in memory, operating as an *aide-memoire*, a keepsake, a testament (Kwint et al., 1999).

Each of my case studies, which originate across the British Isles in the period of 1590–1775, reveals clues to why the family in question placed value on coral for their offspring. Exploring a range of sources, we can take account of their unique family situation while acknowledging common themes of family tragedy, separation, spiritual anxiety and concerns about the survival of the child. Focusing on five families, this article addresses lived experience in three ways. Firstly, I unpack the significance of coral within each case study. While the physical object referred to in family documents is no longer extant or available for analysis in all but one of the case studies, I cross reference between documentary evidence where possible. I also use a reasonable degree of speculation or ‘historical imagination’ to consider how each family’s fears were unique, grounded in their personal situations of heartbreak and persecution (Collingwood, 2014, p. 241; Zemon Davis, 2007, p. 13). Secondly, I analyse coral as an evocative object and a repository of emotion, in line with key works in the fields of the history of emotions and material culture. This method establishes the commonalities in parental experience across the social hierarchy during periods of crisis and uncertainty. Thirdly, I contemplate what might be conveyed in the material language of coral that did not align with the dominant religious, medical or social doctrines of the time, such as Catholic survivalism, interest in the occult, or despair at losing a child to a “better place”, be it Heaven or a benevolent institution.

Given the immense social change within the period, the case studies are presented chronologically to anchor the analysis within a coherent view of the social context. Firstly, I consider the possible concerns of widow, Julian Herrick, who wrote to her deceased husband’s brother to request the transfer of coral from one child to another. Was the coral deemed essential as a teething aid, as protection against the risks of wet nursing outside the family home or as a form of child’s talisman within this family of goldsmiths? The second case study examines the mention of coral in a letter from Sir John Oglander to his wife on the Isle of Wight and elucidates the multiple concerns Sir John may have felt regarding the survival of his young grandson as evident in his commonplace book. The third case delves into Dr Theophilus Garencières’ published account of the significance of the coral bracelet worn by his baby daughter and analyses the role of coral jewellery as a measure of infant health and an agent of memory in tragedy. The fourth example explores the coral heirlooms of Lady Julia Blackett, a descendant of a Roman Catholic family in Northumberland, and investigates how coral passed down to children of the family could be understood within the framework of Catholic devotional culture. Finally, the choice of a coral necklace as an identifying token left with a baby at the Foundling Hospital in London is contextualised within the social and financial situation of the baby’s mother Ann Lord, part of the Spitalfields weaver community. Using these case studies, I suggest that parents understood coral to be familiar and protective within multiple cosmographies. Despite changing religious and medical doctrines, coral continued to embody in material form anxious parents’ greatest hopes and greatest fears for their children. While ostensibly a mere item of children’s jewellery or teething toy, the believed apotropaic and medicinal power of coral as a means of protection and signifier of health served to render it a physical repository of the care and concern parents felt for their children at all levels of the social hierarchy.

## 5 ‘I praie ye send for the courall’: the Herricks of Goldsmith Row

In 1593, Julian Herrick (1561–1629), a widow and mother of six surviving children, wrote from her sister’s home in Hampton to her brother-in-law, Sir William Herrick (1562–1653), the Royal Goldsmith in London.

I praie ye send for the courall from Robard’s nource & send it me for

Wil. I praie dd cõm to you for souch things as is heer set daune … I have sent divers notes be fore to you.^2^

This letter belongs to a collection of fragments of letters from Julian to Sir William. The Herricks were wealthy jewellers from Leicester who had taken up residence in Goldsmiths Row, Cheapside (Cain, 2008). After the suspected suicide of goldsmith and money lender Nicholas Herrick (1542–1592), Julian’s husband and Sir William’s much older brother, Julian moved to Middlesex with baby William and daughter Mercy, while Nicholas’ brothers took custody of the remaining four children including young Robert, aged about 19 months and likely out at wet nurse.^3^ Julian was thus separated from four of her children and seems to have been reliant on Sir William to access certain items for baby William and Mercy.

In her rather pleading letter, Julian was asking Sir William, her sixth child Robert’s guardian, to request the coral from Robert’s nurse and send it to her for her seventh child William. The coral was likely a coral pendant or teether set in silver or gold. *A New Touchstone for Gold and Silver Wares* (1679) by London goldsmith William Badcock included ‘Bells and sockets for corals’ in its title list of ‘Goldsmith Works.’ While we have no way of knowing when the Herricks’ coral was crafted, it might well have been made in Julian’s father’s workshop where Nicholas was apprenticed as a young man or Nicholas’ own workshop where his younger brother William was also apprenticed. Within this family network closely allied to the goldsmith trade, this child’s object may have held a unique place in the family’s history, handed down between the couple’s children in a similar manner to the transfer of *rinkelbels* in the Netherlands.

Baby William was born in 1593 so could only be a few months old at the time of the letter. Nicholas had died shortly before baby William’s birth, undoubtedly casting a shadow over his babyhood and heightening Julian’s anxiety (Clifford, 2004). Julian also had experience of infant loss as her first son, also named William, died in infancy in 1585. Julian’s concern about obtaining the coral for her seventh child may relate to two commonly perceived risks to infant health, wet nursing, and teething.

Young infants were routinely sent out of the Herrick family home for wet nursing, as was fashionable at the time, although children were considered in danger of being ‘overlaid’ or ‘starved’ while at nurse (Fildes, 1988, pp. 93–99). Two of Sir William’s twelve children, born a year apart, died at nurse and were not brought home for their funerals:

My son Thomas (born 3rd May 1602) was nursed at Petersham … lived not long and was buried in the church there … and Elizabeth (born 6th May 1603) was nursed at Highgate … but lived above a year, and died there, and was buried at St Pankers church in the fields by my cozen Toby (William Herrick cited in Fildes, 1988, pp. 82–3)

As it is likely baby William was also sent to a wet nurse, Julian possibly sought the prompt return of the coral for its protective properties. As Musacchio’s research in Renaissance Tuscany reminds us, coral amulets were deemed an essential accessory for infants being wet nursed outside the home where they might face all kinds of natural and supernatural dangers (2005, p. 151). Robert was close to being weaned and Julian may have regarded her youngest son’s need as more pressing.

Alternatively, Julian may have been anxious to prevent serious complications from teething. Coral was used as a teething aid but the medical significance of this cannot be overstated. A coral teether was not simply a teething ‘toy’ (Harris, 2022, pp. 282–3). Teething was regarded as potentially fatal throughout the early modern period. In *The Midwives’ Book*, attributed to Jane Sharp, teething could have dire consequences,

The usual painful Disease of all Children is the Breeding of their Teeth, it is very dangerous to some … If the Teeth be long before they can come forth, Children commonly will die of Fevers and Convulsion Fits (1671, p. 404).

Parisian surgeon, La Vauguion, warned in his surgery manual (translated into English in 1699) that if teething ‘happens for a long time, the Pain is so excessive, that it sometimes kills theChild’ (1699, p. 265). While coral medicine could treat the accompanying fevers and fits, coral teethers were believed to soften the gums to allow the teeth to break through and ease the pain (Blagrave, 1674, pp. 33–4). The bells attached to coral teethers could provide much needed distraction. La Vauguion recommended corals with bells in the first instance but advised surgeons, ‘If the Pain in cutting of the Teeth cause Convulsions, open the Gum with an Incision Knife, going so deep till you feel the Hardness of the Teeth with the Instrument’ (La Vauguion, 1699, p. 266). This was a painful and dangerous remedy in itself, given the risk of accident and infection. Coral teethers and medicines offered parents a first line treatment for their young children, hopeful to avert the need for surgical intervention.

**Figure 2:**
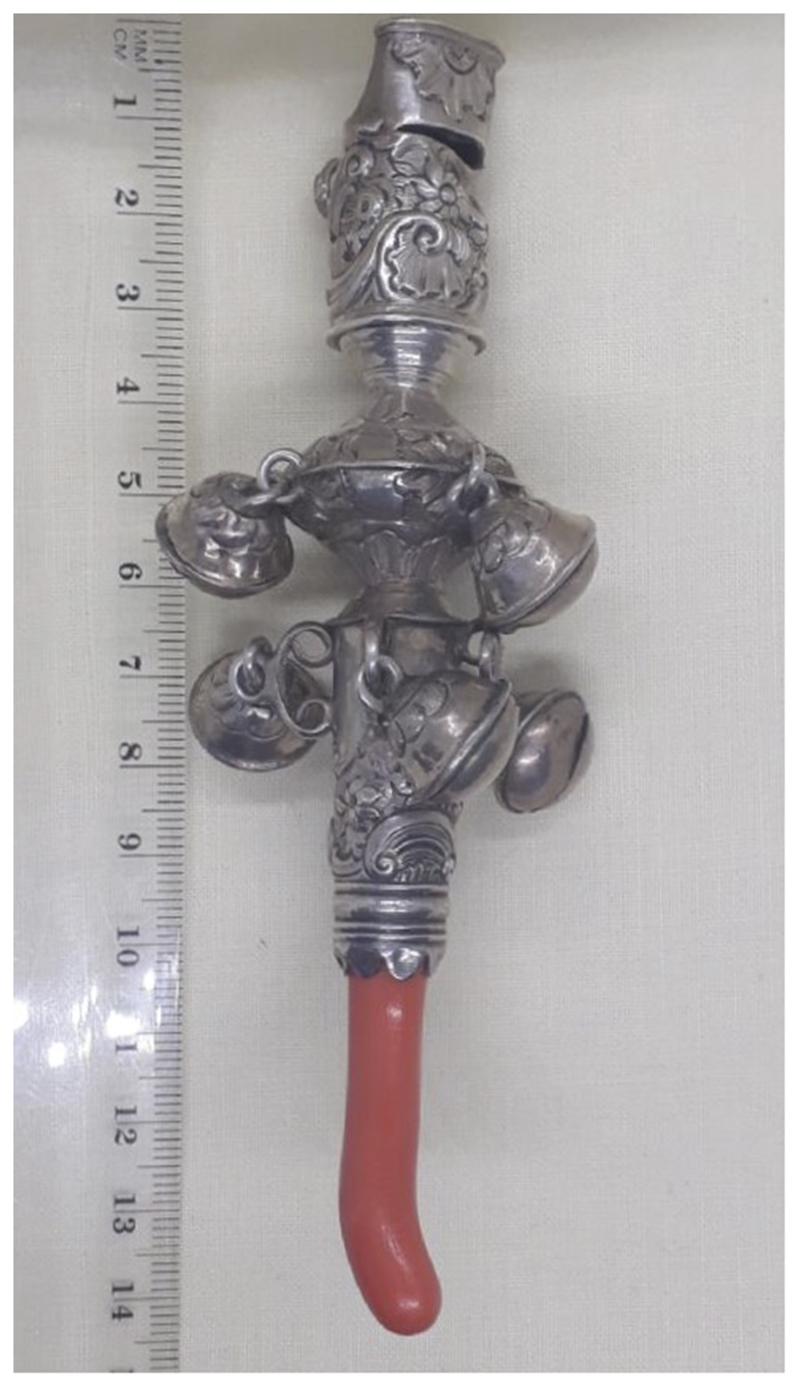
Sandyland Drinkwater (c.1750). Coral and silver teething toy with bells. Author’s own.

Examining children’s material culture offers important complementary evidence to visual and documentary sources and coral teethers record the need to gnaw during an itchy and painful developmental stage. The coral teether in Figure 2 shows bite marks, indicating actual usage by a teething child. The missing bells and bent whistle also bear witness to the unintended uses of the object; perhaps it has been banged and thrown by a toddler who did not respect the quality of the craftsmanship or the expense and significance of coral and silver. These coral teethers were not simply status symbols, depicted in portraits of children to indicate the wealth of their family, but were bought by parents actively seeking to soothe their children’s suffering (Willemsen, 2000, p. 65).

Julian clearly believed that this particular coral was essential for her infant son. In her letter, Julian also requested fabrics to make clothes for William and Mercy and wrote ‘for that you wrote afore time that you hade much beusenes, deliver the monni to mi brother … and I will desire him to provid it me.’ However, a new coral could not be bought for William. Julian desired the family coral which required Sir William’s cooperation. Julian signed the letter ‘your loving sistour’, perhaps in hope of earning his favour.

Indeed, it seems an odd paradox that although Sir William did not attend his own children’s funerals, he had a critical role in transferring children’s coral between family members. The coral was a valuable family heirloom but its value was not purely economic. If we recognise that coral as a material was believed to have apotropaic and health preserving properties for vulnerable infants, ensuring a child had coral with them at nurse could be an act of paternal or avuncular care, albeit from a distance. This is akin to the role of affluent Tuscan fathers who ensured each child was accompanied by coral amulets while separated from the family at nurse (Musacchio, 2005, p. 151). While evidence of infant deaths in the wider Herrick family could indicate coral was an ineffective source of protection from childhood ills, the coral might be understood as a family talisman, a way to send with the child the hope, love and concern of the family embodied within a physical object.

## 6 ‘Kisse him for mee’: the Oglanders of the Isle of Wight

**Figure 3:**
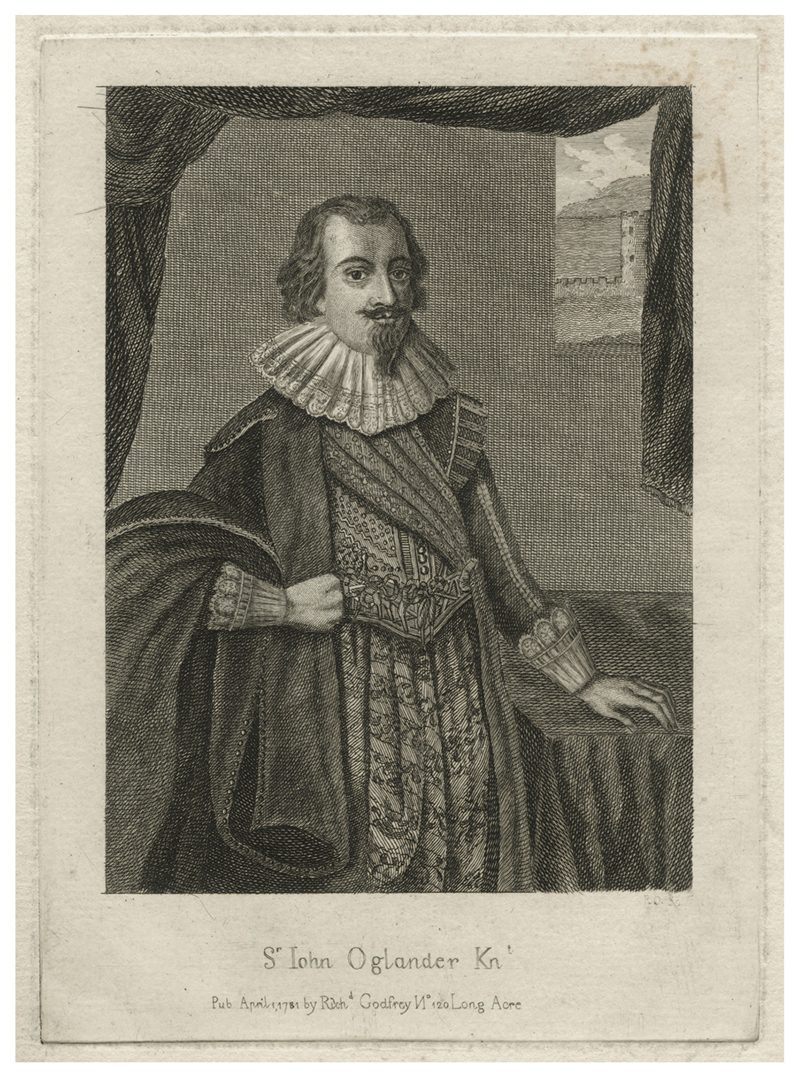
Richard Godfrey (1781). Sir John Oglander, line engraving, [NPG D21549]. National Portrait Gallery, London, United Kingdom.

We move from the urban goldsmiths of London living under the relative stability of Queen Elizabeth’s reign, to the landed gentry of the Isle of Wight facing the Civil Wars of 1642–1651. Sir John Oglander, portrayed in Figure 3, wrote to his wife, Lady Oglander, on 26th July 1643 and, amongst other news regarding the war, asked ‘whether you received Littell Jacke’s Corrall, whom I pray god to Blesse (and kisse him for mee).’^4^ From Sir John’s commonplace book, we can deduce that little Jack was his grandson, godson and name-sake, born on the 15th of March 1642, 12 months after little Jack’s older brother of the same name died. Sir John noted the position of the moon and the hour of his birth, stating that he was ‘born in a miserable, distracted time. God bless him’ (1936, pp. 104–5).

Sir John Oglander, a Royalist, was living in London at the time of the letter mentioning Jack’s coral as he had been called from the Isle of Wight to attend the Parliamentarians’ Committee for Safety. During a difficult time, it is interesting Sir John took it upon himself to purchase this coral for Jack. Why did Sir John perceive a coral to be essential? While it is impossible to elucidate this precisely, we can situate the purchase of coral within the concerns Sir John expressed about seen and unseen threats in his commonplace book.

While Sir John was Jack’s godfather, this was not a baptismal gift as the child was already 17 months old. Did Sir John perceive Jack to be vulnerable to the dangers of teething or the risks of being wet nursed? He was careful about choosing trustworthy local wet nurses, selecting the wives of his shepherd and his tenant to feed his own children (1936, p. 177). It seems unlikely that these were the primary concerns as Jack would have been teething for at least a year and close to being weaned. Sir John may have been anxious about the risks of childhood illnesses. At least three of Jack’s siblings died before the age of five to Sir John’s great sadness, though he does not record the cause of their deaths (1936, p. 108). He did mention the hazards of smallpox affecting families on the island (1936, p. 23) and seventeenth century physicians recommended coral medicines to treat smallpox (Garencières 1676, p. 69, Pechey, 1695, p. 474). However, these would usually be internal medicines, accessible from the island’s apothecary Maynard (Oglander, 1936, p. 111).

Sir John may have felt Jack needed extra protection from occult forces. He had noted that Jack was born at an inauspicious time relating to the moon and the civil war. An interest in astrology was still widespread in the seventeenth century, demonstrated by the popularity of almanacks (Hill Curth, 2017, p. 33). Medical astrologers believed the presence of the moon at birth had particularly profound effects on wellbeing. George Atwell, Professor of Mathematics at the University of Cambridge, wrote in *An Apology or Defence of Astrologie* (1660, pp. 6–7):

Whether experience doth not shew many things in Astrology, even to ignorant people: As every Physician and each Midwife can tell us, that the Child born in the new or full moon is either short lived, or never healthful: and is this unlawful to think it, or judge it to be so?

The moon, *luna*, was also believed to cause imbalances in the moist brain, hence the term ‘lunatic’ and could be associated with idiocy (Thomas, 1973, pp. 351–2). The astrologer Joseph Blagrave (1610–1682) in his *Supplement or Enlargement to Mr. Nich. Culpepper’s English Physitian* (1674, pp. 33–4) stated, ‘Coral is under the dominion of the Sun, yet reputed to be of a cooling, astringent quality: the red the hottest, the white the coldest.’ Coral was regarded as operating on an antipathetic basis; coral as a stone of the sun could counteract the properties of the moon which were believed to be moist and phlegmatic (Blagrave, 1671, p. 6). Blagrave noted that coral indeed ‘cools and dryes up the moisture’ (1674, pp. 33–4). Blagrave recommended using herbs associated with the sun and gathered under the Dominion of the Sun to treat conditions caused by the moon including urinary problems, wind and colic, bronchial catarrh, convulsions and epilepsy (1671, p. 78, 116). It might not be stretching the hypothesis too far to suggest that coral, as a stone under the dominion of the sun, might be given to young children to defend against complaints governed by the moon or to give extra protection to children born during the influence of the moon and thus a particularly unfavourable time.

Sir John may have feared evil spirits on the island jeopardised Jack’s health. Describing the Gardes family of Godhill in his commonplace book, Sir John referred to a ‘notorious’ story of one of the ‘crafty’ brothers who wished to act ‘by the Devil’s means’ and visited a witch living near Ringwood (1936,pp. 151–2). Sir John may have believed a witch posed a threat to the young and vulnerable Jack and coral offered protection. As mentioned by Aubrey, coral amulets were widely believed to defend against the Evil Eye and popular medical texts referred to coral as a deterrent against evil spirits and witches. Culpeper wrote in his translation of the *London Pharmacopoeia* of 1618 that coral ‘helps witchcraft being carried about one’ (1651, p. 35). In *The Store-house of physical practice* (1695), physician John Pechey took the view that coral might help children suffering from night terrors, fits and convulsions, ‘commonly supposed by the ordinary sort of people (to be) occasioned by the Devil, or an Evil Spirit’s lying upon their Stomachs …‘ He stated that coral ‘being hanged about their neck or upon the Pit of the Stomach, may do some good’ (1695, p. 12–13). While referring to the beliefs of ‘the ordinary sort of People’ as separate from the learned physician, Pechey did not rule out the benefits of coral as a delusion like Reginald Scot, instead tolerating popular practices.

For Sir John, as the patriarch of the family seeking to safeguard his descendants, coral offered a means to protect a young child’s health in multiple ways within a single material, without needing to explicitly state what he believed those threats might be. As Sir John’s firstborn son, George, and his firstborn grandson, John, had both died, little Jack’s survival was of huge importance to maintain the Oglander lineage. However, Jack’s position as heir should not bely the love and affection that Sir John had for his family, evident in the request for Lady Oglander to ‘kisse him for mee’. While Sir John was unavoidably detained in London, his own future in doubt and unable to protect his family in person, sending the coral allowed him to express his concern and love in a tangible way. Knowing that his young grandson would be constantly accompanied by the coral, likely suspended on a ribbon around his neck, Sir John may have been reassured that the coral’s apotropaic, prophylactic and curative properties would defend this precious child in his absence.

The Oglanders and Herricks, both gentry families, reveal negotiations about transferring coral to young children during periods of family crisis. In both cases, there is concern regarding obtaining coral for a child whose older sibling and namesake had died. These children were possibly regarded as particularly vulnerable to an array of threats to their wellbeing. In both families, gentlemen were responsible for obtaining coral for their young relatives, either through purchase or securing its return from a wet nurse.^5^ This confirms assertions by authors such as Newton (2012), Evans and Read (2015) and Smith (2006) that male relatives played a role in the wellbeing of young children during this period.

## 7 ‘I came home and spied the coral quite altered’: Dr Garencières of York

Dr Theophilus Garencières (1610–1680), a French born physician, qualified in medicine at the University of Oxford and practised in England for most of his life, at times resident in York and London. In Thw Admirable Virtues, and wonderful effects of the true and genuine tincture of coral, in physick, Garencières recommended coral tincture as a cure for many diseases including smallpox and plague (1676, p. 69). He also described how the changing colour of his daughter’s coral bracelet provoked his anxiety both as a father and a physician.

It is an undoubted truth That Red Coral will grow pale, blewish, and maculated with several spots when it is worn by someone who is nigh death, or dangerously sick, and will foretell Disease by the changing of its colour. This I found true by a sad experience of my own; for having once a Girl about Twelve Moneths old that wore a bracelet of Coral, she fell into a Pestilential Feaver: So that I came home and spied the Coral quite altered, I began presently to despair of her recovery … She lived but two days in that case. After her death I would have taken the Bracelet from her and tried whether I could have brought it to its former colour again; but the Mother would never suffer it, but would have it buried with her, lest the sight of it should bring her into remembrance of her loss.

In this tragic account, this father believed that the red quality of the baby’s coral bracelet offered reassurance whereas the coral’s change of colour raised concern that this contagious illness would be fatal. The coral bracelet was so emotionally linked with the young child, as a constant adornment and signifier of health, that when the child died her mother could not bear to keep it as a memento but decided it must accompany the child into her grave.

The redness of the coral to indicate health was associated with the child’s own rosy, robust complexion and when the child grew pale and weak, the colour of the coral changed also. Leah Astbury notes that newborns’ skin tone was carefully assessed (2007, p. 86). A red baby, crying lustily at birth, was believed to have a much better chance of survival than a pale, wan infant. As Dr Pechey wrote on newborns in his *General Treatise on the Diseases of Infants and Children* (1697, pp. 1–2),

It is best when the colour is reddish all over the Body, for that by degrees turns daily florid: but those Children that are at first Florid or White, are most commonly of an ill temperament, Cold, Dull, and not long lived.

The natural colour of coral then lent it agency as a material that shaped the perception of parents. As an indicator of health, coral enhanced or gave credence to the parent’s own observations of their child’s well-being. The pinky-red of the coral matched beliefs in the healthiness of a ruddy complexion, and a change in colour in the coral affirmed the parent’s suspicion that the paleness of the child was a reason for concern. Rubies were also described as having this power to indicate health and sickness, reflecting the significance of the colour red. In *The Faithful Lapidary*, Thomas Nicols wrote that a ruby ‘keepeth the body in safety, and that if any danger be towards it, it will grow black and obscure, and that being past, return to its former colour again’ (1659, p. 58). However, coral was a much more affordable stone and traditionally associated with children.

The coral bracelet was also an ‘evocative object’ to use Turkle’s phrase (2011, p. 5). When the child died, Dr Garencières reported that her mother feared the coral bracelet, once evoking youthful promise, would now only elicit the distress of remembrance, serving to remind her of the child and the tragedy of her premature loss. For the mother the object, so implicated in the child’s wellbeing as a signifier of both vitality and decline, was transformed from an indicator of health to an agent of memory. The memory of the child and her fatal illness located in the coral bracelet might evoke an emotional response which could prove unbearable. Rather than the usual custom of keeping a treasured object as a substitute for an absent person, the act of burying the coral bracelet sought to bury the memory of the child with her.

## 8 A coral and a coral necklace: Lady Blackett of County Durham

Leaving this tragic case, we move to an affluent Northern family in the eighteenth century. In 1718, Sir Walter Calverley, 1st baronet (1670–1749) of Yorkshire recorded in his diary that his mother-in-law, Lady Blackett, gave a coral and a coral necklace to his infant son, Watty (1886, p. 119).^6^ This coral, presumably a pendant, branch or teether, and the coral necklace had been worn by Lady Blackett in her infancy. Lady Julia Blackett née Conyers (1668–1722) of Newcastle was the mother of 10 children who each took a turn with the coral heirlooms before they were passed to her grandson. Along with the coral items, Lady Blackett sent ‘5 guineas to buy him a coat’ (Calverley, 1886, p. 119). As in the case of the Herricks, clothing could be bought but a child’s coral must be gifted within the family.

Lady Blackett had a strong Catholic influence in her heritage and the counties of Yorkshire and Northumberland were known Catholic strongholds since the Reformation (Haigh, 1981). Lady Blackett’s mother, Julia Lumley (1611–1691), was born at Lumley Castle near Durham (Kirtley et al., 2007–2019).^7^ The Lumleys of Lumley Castle were well known in the area as devout Catholics and, while locally respected, were implicated in treacherous dealings against the Crown including the Pilgrimage of Grace and Ridolfi plot (Milner, 1904; Newman, 2004).^8^ The Lumleys continued to practice Catholicism in the seventeenth century, Lady Blackett’s first cousin Richard Lumley, the first Earl of Scarborough (1650–1721), was raised a Roman Catholic and went on a Grand Tour with his Confessor, Richard Lassels (Childs, 2004). While we do not know if Lady Blackett herself was a practising Catholic, if we consider the importance of the coral heirlooms to Lady Blackett in conjunction with her Catholic family connections and the significance of coral within the Catholic Church, the provenance of her coral and coral necklace is fascinating to explore.

Imported red coral beads were a popular material for Catholic rosaries in England pre-Reformation (Armstrong, 1973, p. 69). Rosaries were an essential part of everyday Catholic devotional culture; rosary beads ‘were animated and became the vehicles of spiritual experiences and the repositories of memory’ (Galendra-Cooper & Laven, 2017, p. 350). Rosaries themselves were understood to hold a special apotropaic power. Intended as a physical *aide-memoire* while reciting the Marian psalter, they were closely associated with the Virgin Mary who was believed to be a compassionate mother, able to intercede on behalf of sinners on Judgement Day (Heal, 2007, p. 23). Imbued with the Virgin’s power, rosaries offered protection from illness and evil spirits and would be worn at night or clasped on the death bed (Winston-Allen, 1998, p. 116). When the Statute of the Treason Act of 1571 banned the import of Catholic devotional items including crosses, pictures and beads to England, coral rosaries may have been carefully hidden by devout Catholic families.^9^ It is possible that Lady Blackett’s coral beads were handed down from her mother Julia Lumley, originating as a rosary owned by the Lumley family and restrung as a child’s necklace. These coral beads may have prompted memories going back through generations, a physical reminder of now illicit practices such as learning the catechism on rosaries at their mother’s knee (Sanders, 1998, pp. 1–22) or holding them at night for protection.

**Figure 4:**
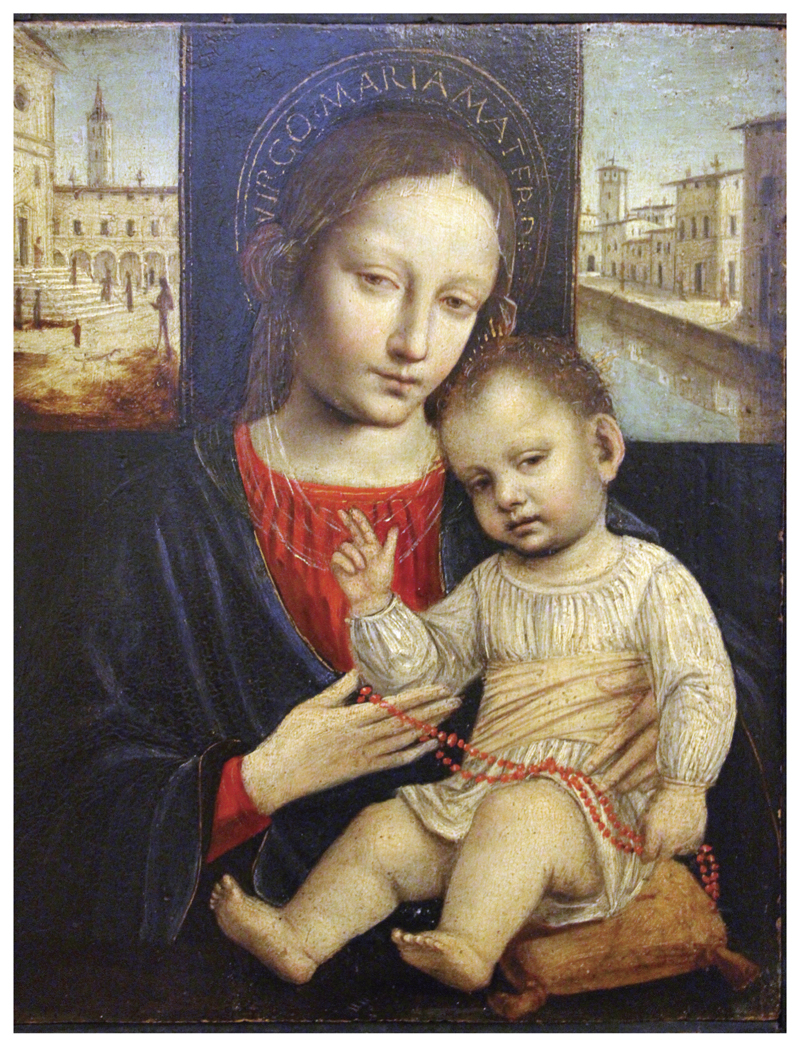
Ambrogio Bergognone (c.1500–1510), Madonna and Child, Oil, Musée Poldi Pezzoli, Milan, Italy. http://wikiart.org/en/ambrosio-bergognone/madonna-and-child-1510 (public domain).

**Figure 5:**
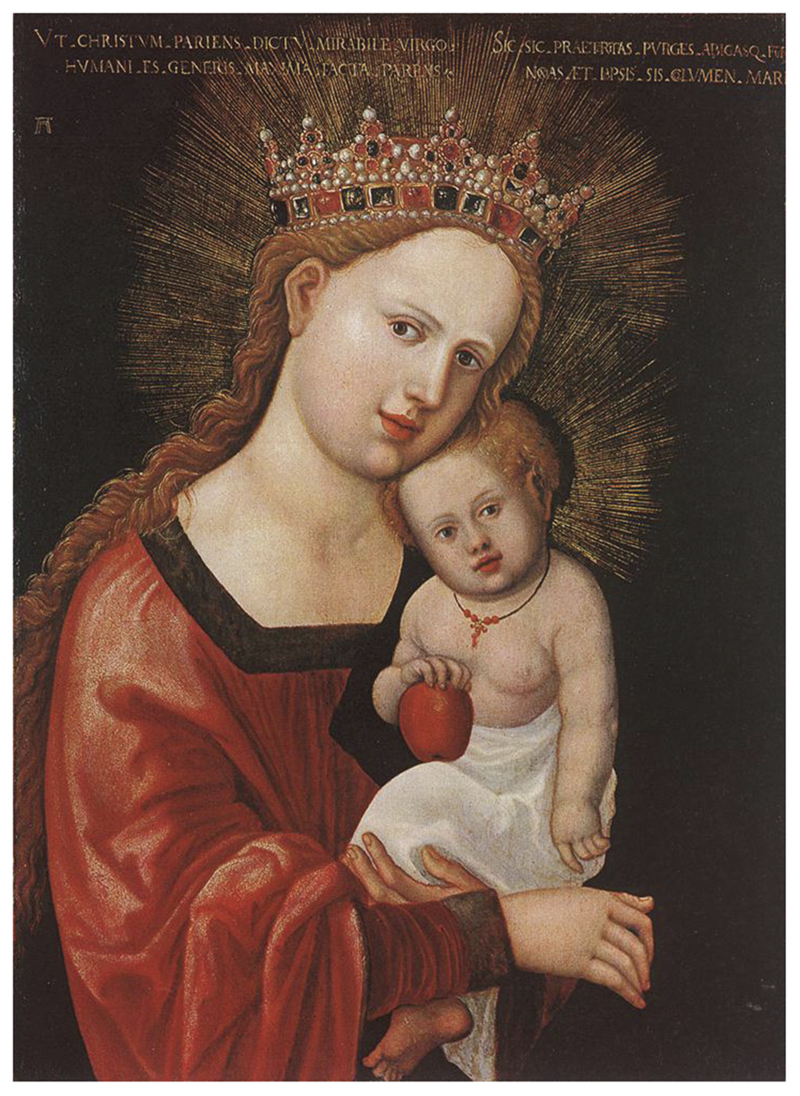
Albrecht Altdorfer (c.1520–25), Mary with the Child, Wood, Museum of Fine Arts, Budapest, Hungary. http://www.wikiart.org/en/albrecht-altdorfer/mary-with-the-child-1525 (public domain).

Red coral was also associated with infancy in Catholic visual culture. Depictions of the Christ Child wearing or playing with coral beads appeared in Catholic iconography across Europe. In Figures 4 and 5, portraits by Italian artist Ambrogio Bergognone (c.1470s-1523/24) and German artist Albrecht Altdorfer (1480–1538) depict the Christ Child with coral beads. In these portraits, the Madonna and Christ Child are portrayed in an idealised loving embrace; the child with a coral necklace nestled on his mother’s lap while both engage the viewer with a sentiment of contentment and natural intimacy (Wise, 2013, p. 27). Although little English devotional art survives from this period due to the programme of iconoclasm during the English Reformation, the interconnectedness of Catholic devotional culture via the See of Rome likely rendered the association between coral and the Christ Child familiar in Catholic England. It has been suggested that the scarlet of the coral beads was intended to act as a foreboding signifier of the blood of Christ’s passion (Endt-Jones, 2013, p. 9; Musacchio, 2005, p. 151; Pointon, 2010, p. 135; Wise, 2013, p. 26). Yet its presence in playful depictions of the Christ Child in images where his mother the Virgin is caring for him could represent the parental bond and loving protection, rather than a harbinger of death. Joe Moshenska suggests that play could be viewed as a pious activity within Biblical texts and a playful infant Christ was seen as imbuing the joy of his birth and presence as a living God (2019, p. 46). Indeed, playing with religious objects might increase their potency rather than reduce it (2020, p. 161). It could be argued that the Christ Child transmits his holiness to the material he plays with, rendering coral sacred and worthy as a gift to infants everywhere.

Placed within this context, the handing down of coral heirlooms within a family with Catholic connections in Post-Reformation England holds deep significance. For Lady Blackett, watching her children and grandchildren wearing and playing with coral beads may have epitomised, if not a form of closet Catholic piety, at least a positive association with the Christ child of Catholic iconography. Lady Blackett may also have sought to invoke the protection of the Virgin Mary, as the coral was handed down the female line and imbued with maternal power. Lady Blackett’s daughter, Julia, had only two children, baby Watty was born in 1707 followed by a sister, Julia, seven years later (Kirtley et al., 2007–2019). As a mother of ten children herself, Lady Blackett may have been concerned about the fertility of the marriage and the gift of the coral heirlooms might have reassured an anxious grandmother seeking to safeguard these precious grandchildren.

The presence of coral jewellery as an heirloom may have broader religious implications. In Protestant England, recusants could easily secrete rosaries on their person but they would have incurred a high penalty if discovered (Walsham, 2014, p. 376). The re-stringing of precious coral beads as children’s jewellery effectively hid them in plain sight and were believed to infer special protection on the children who wore them. I would argue that coral retained its protective qualities within its material form, allowing families, anxious about maintaining their faith and living a pious life, the opportunity to discreetly keep devotional items within the family. By gifting coral to children, this coral symbolised the hopes and fears regarding the both survival of the children and the survival of the faith. Perhaps Julia Calverley, named for her mother, grandmother, and great grandmother, became the next owner of the coral heirlooms to pass to future generations.

## 9 A foundling token: Ann Lord of Bethnal Green

**Figure 6:**
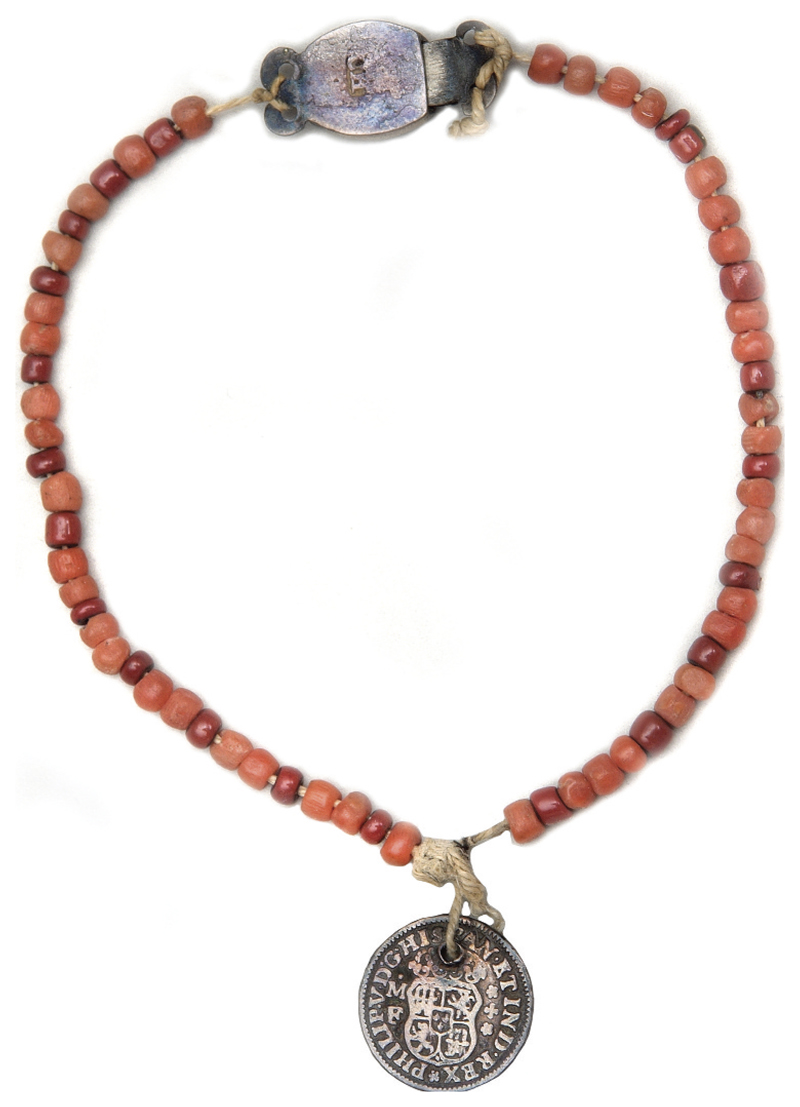
Unknown artist (n.d.). Coral Necklace with Spanish coin pendant left by Ann Lord with child number 77,038 in 1775, © the Foundling Museum, London, United Kingdom.

Amongst hundreds of tokens at the Foundling Museum in London, we find this coral necklace shown in Figure 6. Deserted by her baby’s father, a journeyman weaver, Ann Lord petitioned for a place for her young daughter at the Foundling Hospital in 1775; she left the coral necklace with her baby as an identifying token (Bright & Clarke, 2014, p. 19). In the eighteenth century, it was customary to leave scraps of fabric, notes or trinkets as identifying tokens so parents could reclaim the child in the future. Some of these trinkets were quite financially valuable. Coral was still highly prized in this period; in 1767 William Plush declared that a coral necklace with silver locket attached was stolen from the neck of his son James, aged nearly three years old, in Borough Market in Southwark.^10^ The existence of coral necklaces as tokens at the Foundling Hospital suggests that desperate parents chose not to sell their child’s coral necklace but felt it important their child’s coral went with them, not to their grave in the case of Garencières’ daughter but into an institution. Infants were re-named and fostered out to wet nurses by the Hospital and most were never reunited with their parents due to high mortality rates or because their parents were never able to afford the re-claim fee (Bright & Clarke, 2014). Leaving a coral token which might offer some protective benefit could be the last act of care and love a parent was able to give their child.

In 1772, three years before Ann gave up her daughter, the Hospital began to give receipts to mothers to reclaim their children rendering tokens unnecessary. We can therefore interpret Ann’s decision to leave the coral necklace with her baby as particularly important. John Styles (2010) and Maria Zytaruk (2015) have both analysed the deeper significance of tokens within the Foundling Hospital collection. While Styles focuses on textile tokens, he writes that the choice of objects consisting of specific materials, decorated with particular colours or emblems, was highly symbolic in a world where materials acted as a form of communication to convey meaning. Thus ‘material literacy’ not only supplemented verbal literacy but could replace written literacy where skills were limited (2010, p. 70). In the case of tokens, the choice of token could be intended to communicate respectability, emotion, or a desire to be reunited. Like Styles, Zytaruk perceives the tokens to act within a material *lingua franca* but goes further to argue that a token, in the way of an elegy, acted to mark a moment of loss and grief and console the bereaved, both the mother and child (2015, p. 339). Zytaruk argues that more unusual tokens might suggest a deeper emotional investment, writing that ‘mothers looked to objects to materialise the experience of thwarted love’ (2015, p. 332). There are only a very small subset of coral necklaces, bracelets and teething beads within the Foundling Museum collection and analysing the case of a coral necklace and “I.W” pendant left with a two-month-old baby boy in 1754, she suggests that coral tokens became ‘surrogates for a mother’s presence’, chosen to protect, comfort and soothe due to their amuletic and analgesic properties (2015, pp. 335–6).

We can explore Ann Lord’s choice of a coral necklace for her daughter by contextualising her decision within her own personal circumstances, the wider social situation and the demands of the hospital’s admissions criteria. While admission procedures changed over time, from 1763–1801 impoverished parents could petition for a place for their infant and the mother’s ‘moral character’ became an important criterion of the child’s acceptance (Howell, 2014, pp. 20–22). Ann petitioned for her child’s place and had to prove her respectability in a highly competitive application process. Thanks to the research of Janette Bright and Gillian Clarke, we know that weavers living in Bethnal Green vouched for Ann as a woman of honest reputation. Mr James Godline of Tyson Street acted as a character reference and Mr and Mrs Simpson of Club Row stated that they had known Ann since she was a child and that she was respectable and hardworking (2014, p. 19). Given that two weavers vouched for Ann, Mr Godline and Mr Simpson, and the father of her baby was listed as a journeyman weaver, it is likely Ann grew up as part of the close-knit weaving community of Spitalfields and the adjoining parish of Bethnal Green. As Peter Burke has noted, artisan communities in England and across Europe shared a particular history and culture built on a system of apprentices, journeymen and masters, and during the latter half of the eighteenth century the weaving trade in Spitalfields, originally established by Huguenots, was undergoing particular upheaval (2016, pp. 64–72). There were frequent riots in the 1760s and the first Spitalfields Act of 1773 was passed two years before Ann gave up her baby in 1775. The Act was intended to reduce the rioting and stated that magistrates should set standard rates of pay. This prevented Masters rewarding talented craftsmen and paying lower rates to workers in quiet periods or to old or disabled employees who needed the work to survive (Clapham, 1916, pp. 60–6). A clause also prevented Masters from employing weavers not resident in the Spitalfields area. The increasing unprofitability of weaving in Spitalfields and difficulties obtaining work by non-resident weavers may have precipitated the moving on of Ann’s baby’s father, an itinerant weaver, to pastures new leaving Ann in an even more financially precarious position. The coral necklace along with the gold coin was a valuable item, possibly constituting vital collateral in arguing Ann’s respectability in this particular period of social unrest.

Ann may have chosen to leave coral due to enduring beliefs in its amuletic properties. Possibly unaware that the hospital would remove all items from the child and store the necklace with the child’s paperwork, Ann may have hoped the coral necklace would stay around her child’s neck as a protective charm. Even if she had been aware of the physical separation of token and child, she might have believed that the coral would continue to protect the child as it was held by the same institution charged with the child’s care. As we have seen in the previous case studies, parents seem to have had an emotional investment in their child’s coral, believing it to be of huge importance that it accompanied the child especially through periods of separation. In this case, the coral necklace may have acted as a physical repository for Ann’s love for her daughter. Possibly illiterate and unable to write or embroider a note expressing her love, this material object could convey the emotional attachment that Ann herself could not document. This coral necklace may have been purchased by Ann for this express purpose or been a treasured heirloom within Ann’s own family, transmitting maternal love down generations.

While we can never know Ann’s true motivations in choosing a coral necklace as an identifying token, we can juxtapose this choice alongside what she did not choose. Indeed, it was not obligatory by this stage in the hospital’s history to leave a token at all. As a member of the weaving community, Ann could have chosen a scrap of silk as a material which would convey a sense of professional identity or a link to the baby’s father, alongside many other textile tokens left during this period. She could have chosen to leave the Spanish gold coin without the coral necklace. Or a note written by a friend. Zytaruk has suggested that the choice of a necklace worn by the infant and recorded by hospital officials as ‘marks on the body’ constituted ‘a form of inscription - writing on the body of the child’ (2015, p. 334). In this sense, we might understand the coral necklace as a very personal form of communication, writing Ann’s hopes and fears on the body of her infant daughter, marking a moment of loss and anxiety and also yearning for a better future by drawing on red coral’s symbolism of health and protection. In this case, Ann’s daughter Mary survived and at age ten was apprenticed to a goldsmith.

## 10 Conclusion

Julian Herrick, a goldsmith’s widow, Sir John Oglander, a knight caught up in the civil war, Dr Garencières, a doctor confronting dangerous infection, Lady Blackett, a devoted grandmama, and Ann Lord, a woman facing destitution, all placed value on coral for their young children. From these case studies, it can be argued that coral operated within wider kinship networks as an amulet, a devotional material, a medical aid and a token of attachment and memory, reflecting the anxieties of mothers, fathers and grandparents across the social hierarchy and across England regarding infant loss. In a diverse political, religious and medical landscape, children’s coral could speak within multiple frameworks without the need for written and potentially incriminating documentation.

The enduring practice of giving young children coral in early modern England thus poses questions about the beliefs of parents living in complex physical, spiritual and emotional worlds. Analysis of the sources presented here has attempted to understand those worlds in which parents might fear a multitude of natural and supernatural threats associated with infection, disease, pain, the untimely movement of celestial bodies, evil spirits or separation. While this article has explored possible interpretations of these sources using an expansive approach, there is of course the potential for further research to support or challenge these ideas.

I have argued that coral can be understood as the material embodiment of the anxieties of parents and grandparents and also a material expression of hope, affection and faith, particularly during periods of crisis and separation. By drawing on material approaches and the history of emotions, we can identify coral as part of a non-verbal language at a time when materials were a means to convey much more than they do today. As we have seen within these diverse case studies, decoding this language is not a simple process. Red coral presents a unique view of parental emotion in the early modern period because it reflects the varied and enduring cosmographies at play within a single material.

## Notes

1 I use the term ‘occult’ in terms of a general and popular understanding of forces which acted in hidden or inexplicable ways, such as evil spirits, The Evil Eye and witchcraft. For more on occult philosophy, see F. Yates (2001).

2 Bodleian Library, letter, [MS.Eng.hist.c.474/fol.110]. Letter from Julian Herrick to Sir William Herrick, May 1593. My thanks to Anna-Nadine Pike for her assistance locating this letter.

3 Leicester Records Office, Herrick Manuscripts, DG 9/2413, Agreement.

4 Isle of Wight Record Office, letter, OG/CC/53, Letter from Sir John Oglander, London to His Wife, Frances Oglander, July 26th, 1643.

5 For an example of an eighteenth-century grandfather gifting coral to a male heir, see E. Foyster (2016, p. 11).

6 This diary entry is also cited in (Anne Buck, 1996, p. 59). This Walter Calverley is not the same Sir Walter Calverley of Yorkshire (1570–1605) infamous for murdering his two young children.

7 Lady Blackett’s father, Sir Christopher Conyers (1621–1693), is not known to be Catholic, other Conyers in the area were listed as recusants (Hilton, 1977, pp. 53–4) and previous intermarriage occurred between the Lumleys and Conyers (Newman, 2004).

8 John Lumley, 5th Baron Lumley (1493–1544) was involved in the rebellion against Henry VIII’s break with the Catholic Church, The Pilgrimage of Grace, in 1536 and his son, George, was executed for his role in the second Pilgrimage of Grace in 1537 (Newman, 2004). His grandson, Sir John Lumley, 1st Baron Lumley, (1533–1609), was suspected of collaborating with Mary Queen of Scots (Milner, 1904, p. 53). When he died without issue, Lumley Castle passed to his cousin, Richard, 1st Viscount Lumley, Lady Blackett’s maternal grandfather.

9 13 Eliz. I c.2. See Statutes of the Realm, 4 vols (London: HM Stationery Office, 1819), 4.1: 530–1.

10 Surrey History Centre, Surrey Quarter Sessions, QS2/6/1767/Mic/26, Richard Wheeller and Isaac Long Accused of Stealing a Coral Necklace and Silver Locket from the Neck of James Plush Aged nearly Three Years in the Borough Market, Southwark.
